# Supplementary material for: A Critical View of the Application of the APEX Software (Aqueous Photochemistry of Environmentally-Occurring Xenobiotics) to Predict Photoreaction Kinetics in Surface Freshwaters
Source: Molecules. 2019 Dec 18;25(1):9. doi: 10.3390/molecules25010009 (PMC7017383; doi:10.3390/molecules25010009)
Supplement: Supplementary file 1 [file molecules-25-00009-s001.zip › 3.2.4_gcc-4.4.0.zip!/3.2.4_gcc-4.4.0/3.2.4_gcc-4.4.0/doc/octave/html/interpreter/Stem-Series.html]

Stem Series - Untitled


Next: Surface group,
Previous: Stair group,
Up: Object Groups


---

##### 15.2.8.10 Stem Series

Stem series objects are created by the `stem` or `stem3`
functions. Each `hggroup` element contains a single line object
as a child representing the stems. The properties of the stem series
are

`showbaseline` `baseline` `basevalue`: The property `showbaseline` flags whether the baseline of the stem series is displayed (default is "on"). The handle of the graphics object representing the baseline is given by the `baseline` property and the y-value (or z-value for `stem3`) of the baseline by the `basevalue` property. Changes to any of these property are propagated to the other members of the stem series and to the baseline itself. Equally changes in the properties of the base line itself are propagated to the members of the corresponding stem series. `color`: The RGB color or color name of the line objects of the stems. See Colors. `linewidth` `linestyle`: The line width and style of the line objects of the stems. See Line Styles. `marker` `markeredgecolor` `markerfacecolor` `markersize`: The line and fill color of the markers on the stems. See Colors. `xdata` `ydata` `zdata`: The original x, y and z data of the stems. `xdatasource` `ydatasource` `zdatasource`: Data source variables.
